# Adiponectin levels among individuals with varied employment status in Japan: a cross-sectional analysis of the J-SHINE study

**DOI:** 10.1038/s41598-019-47448-2

**Published:** 2019-07-29

**Authors:** Hoichi Amano, Yoshimi Shirakawa, Hideki Hashimoto

**Affiliations:** 1 Graduate School of Public Health, Teikyo University, Tokyo, Japan; 2 Department of Health and Social Behaviour, School of Public Health, The University of Tokyo, Tokyo, Japan

**Keywords:** Cardiology, Obesity, Epidemiology

## Abstract

The purpose of this study was to examine the association between employment status and adiponectin levels. This cross-sectional study was a part of the Japanese Stratification, Health, Income, and Neighborhood study, a population-based survey in metropolitan Japan. The analysis included data from 848 individuals. A one-way analysis of variance was used to assess differences in log-transformed adiponectin levels among individuals according to their employment status. Multiple linear regression analysis was used to assess these differences after adjusting for other cardiovascular disease risk factors. The main outcome was log-transformed adiponectin. Of the participants, 6.2% of the men and 15.1% of the women were precarious workers. Mean adiponectin values differed significantly by employment status in men, but not in women. In men, multiple regression analysis showed that precarious workers had significantly lower adiponectin levels than permanent workers (β = −0.16, P = 0.02). However, in women, adiponectin levels were significantly lower only in precarious workers with low household incomes (β = −0.35, P = 0.02). Male precarious workers and their female counterparts with low annual household incomes had significantly lower levels of adiponectin. These results might help us to understand mechanisms underlying the relationship between employment status and cardiovascular disease.

## Introduction

Previous research has found significant associations between precarious work status and risk of obesity, hypertension, diabetes mellitus^1,2^, cardiovascular disease^3^, poor mental health status^4^, and higher mortality^5–7^. Precarious workers have a high-risk of cardiovascular disease; however, the underlying reasons for this are not completely understood.

Adiponectin, an adipocytokine, is produced by, and secreted from, adipocytes. Blood levels of adiponectin decrease with the accumulation of visceral fat, so adiponectin is thought to have a beneficial effect. There is evidence that adiponectin plays an important role in chronic inflammation, oxidative stress, insulin-sensitizing, and atherosclerotic processes^8^. In contrast to most adipokines, adiponectin secretion is decreased in obese individuals^9^. Previous observational studies have shown that lower levels of adiponectin are associated with hypertension, diabetes, hyperuricemia, metabolic syndrome, and cardiovascular disease^10-12^. In contrast to these findings, higher levels of adiponectin have been shown to be associated with increased all-cause mortality rates and cardiovascular mortality rates^13,14^. However, as several epidemiological studies have found that adiponectin levels have an inverse relationship with metabolic diseases and arteriosclerosis, factors that increase adiponectin levels are still considered to an indicator of metabolic diseases and atherosclerosis

In addition, some observational studies have shown that socioeconomic status is associated with adiponectin levels^15^. However, the association between employment status and adiponectin levels is unclear. Investigating this association may help us to understand potential biological pathways linking employment status and cardiovascular disease. Thus, the purpose of this study was to assess the association between employment status and adiponectin levels.

## Results

### Participant characteristics

The characteristics of the study participants are shown in Table 1. Of the men, 79.7% were permanent workers and 6.2% were precarious workers. Of the women, 36.7% were permanent workers and 15.1% were precarious workers.

**Table 1:** Characteristics of the study participants.

	Men (N = 404)	Women (N = 444)
Age (years)	38.2 (7.1)	37.1 (7.3)
Waist circumference (cm)	86.5 (8.9)	78.2 (12.6)
Systolic blood pressure (mmHg)	123.6 (18.2)	111.6 (17.9)
Diastolic blood pressure (mmHg)	79.6 (12.8)	71.6 (11.4)
**Marital status**		
Married (%)	76.7	65.3
**Employment status**		
Permanent (%)	79.7	36.7
Precarious (%)	6.2	15.1
Other (%)	14.1	48.2
**Job stress**		
Effort-Reward Imbalance score	0.74 (0.14)	0.66 (0.15)
**Psychological stress**		
Kessler Psychological Distress Scale (K6) score	20.0 (4.6)	19.9 (4.4)
**Lifestyle**		
Smoking status		
Never (%)	37.0	67.2
Former (%)	31.2	21.4
Current (%)	31.8	11.4
Physical exercise		
None (%)	37.7	40.9
Light exercise more than once a week (%)	20.8	17.8
Heavy exercise once or twice a week (%)	36.5	34.5
Heavy exercise more than three times a week (%)	5.0	6.8
**Socioeconomic status**		
Years of education		
More than 12 (%)	67.9	56.8
Household income (million JPY/year)		
Less than 2.99 (%)	7.8	50.7
3.00–7.49 (%)	40.2	41.7
More than 7.50 (%)	52.0	7.7
**Blood biochemistry**		
LDL-C (mg/dl)	106.2 (28.9)	98.8 (31.3)
HDL-C (mg/dl)	60.0 (17.3)	71.4 (22.0)
HbA1c (%, NGSP)	5.2 (0.5)	5.1 (0.5)
Adiponectin (μg/dl)	5.5 (3.1)	9.2 (5.1)
Log (adiponectin)	0.66 (0.28)	0.89 (0.28)

Note: The results are expressed as means ± standard deviations, or as percentages.

LDL-C: low-density lipoprotein cholesterol; HDL-C: high-density lipoprotein cholesterol; HbA1c: haemoglobin A1c; NGSP: National Glycohemoglobin Standardization Program; Log (adiponectin): log-transformed adiponectin.

### Adiponectin levels according to sex and employment status

**Figure 1:**
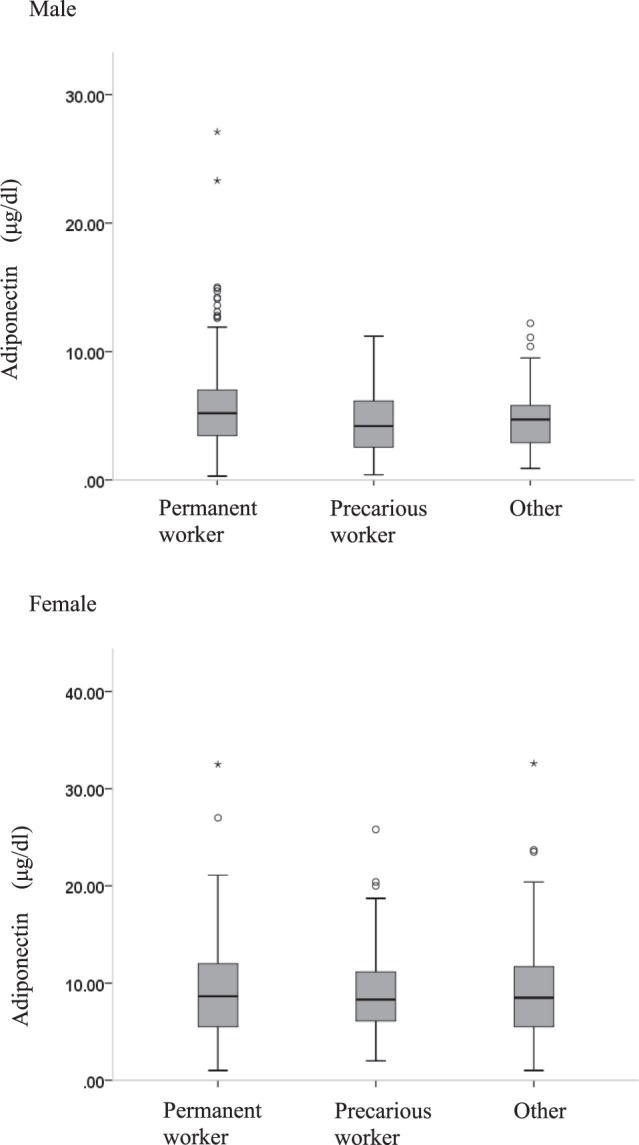
Values of adiponectin by employment status.

The unadjusted values of the adiponectin levels by employment status are shown in Fig. 1. Table 2 shows that the mean log-transformed adiponectin value differed significantly by employment status among men (P = 0.03), but not among women (P = 0.66). In males, precarious workers had significantly lower levels of log-transformed adiponectin than permanent workers.

**Table 2:** Values of log-transformed adiponectin by employment status.

	Permanent worker		Precarious worker		Other		ANOVA P-value
	Mean	SD	Mean	SD	Mean	SD	
Men							
Log (adiponectin)	0.68	0.27	0.54*	0.39	0.62	0.27	0.03
Women							
Log (adiponectin)	0.90	0.27	0.90	0.24	0.88	0.30	0.66

Abbreviations: ANOVA: one-way analysis of variance; Log (adiponectin): log-transformed adiponectin; SD: standard deviation.

*Male precarious workers had significantly lower levels of log-transformed adiponectin compared with male permanent workers.

### Factors associated with adiponectin according to sex

Table 3 shows the results of the multiple linear regression analysis of log-transformed adiponectin. In males, Model 1, which adjusted for age, found that precarious workers had significantly lower levels of log-transformed adiponectin than permanent workers (β = −0.11, P = 0.03). The results remained statistically significant after adjusting for age, systolic blood pressure, marital status, smoking status, physical exercise, years of education, annual household income, job stress, psychological stress, and blood biochemistry (Model 2; β = −0.16, P = 0.02). In contrast, in females, there was no significant association between employment status and the log-transformed adiponectin levels in the adjusted models.

**Table 3:** Standardised coefficients for log-transformed adiponectin using multiple regression analysis.

Employment status	Model 1*			Model 2^†^		
	β	SE	P-value	β	SE	P-value
Permanent	Ref			Ref		
Men						
Precarious	−0.11	0.57	0.03	−0.16	0.08	0.02
Other	−0.07	0.04	0.16	−0.05	0.06	0.56
Women						
Precarious	−0.03	0.04	0.65	−0.01	0.05	0.93
Other	−0.05	0.03	0.32	−0.001	0.05	0.99

*Model 1: Adjusted for age.

^†^Model 2: Adjusted for age, waist circumference, systolic blood pressure, marital status, smoking status, physical exercise, years of education, annual household income, job stress, psychological stress, low-density lipoprotein cholesterol, high-density lipoprotein cholesterol, and haemoglobin A1c.

Abbreviations: β: standardised coefficient; Ref: reference group; SE: standard error.

### Associations between adiponectin and job status in women, according to household income level

Table 4 shows the standardised coefficient calculated through multiple regression analysis for log-transformed adiponectin among female workers stratified by their level of annual household income. There was a significant interaction between employment status and annual household income among female workers (P = 0.07). In females, precarious workers with a household income less than 2.99 million JPY/year had significantly lower levels of log-transformed adiponectin, than permanent workers in the adjusted analysis (β = −0.35, P = 0.02).

**Table 4:** Standardised coefficients for log-transformed adiponectin values in women, according to their employment status and annual household income.

Household income (million JPY/year)	<2.99			3,00–7.49			≥7.50		
Employment status	β	SE	P-value	β	SE	P-value	β	SE	P-value
Permanent	Ref			Ref			Ref		
Precarious	−0.35	0.11	0.02	−0.01	0.05	0.92	0.02	0.05	0.76
Other	−0.05	0.10	0.73	—	0.04	0.62	0.01	0.04	0.89

P interaction = 0.07.

Adjusted for age, waist circumference, systolic blood pressure, marital status, smoking status, physical exercise, years of education, job stress, psychological stress, low-density lipoprotein cholesterol, high-density lipoprotein cholesterol, and haemoglobin A1c.

β: standardised coefficient; Ref: reference; SE: standard error.

## Discussion

The purpose of this study was to examine the association between employment status and adiponectin levels. We found that male precarious workers, as well as their female counterparts with low annual household incomes, had significantly lower adiponectin levels than did permanent workers.

There are several reasons why employment status might influence adiponectin levels. The first possible reason is that food intake may be linked to adiponectin levels. In some studies, omega-3 fatty acids, magnesium, cereal fibre, and a high-fat diet were significantly associated with circulating adiponectin. A systematic review and meta-analysis of randomised controlled trials, found that fish oils such as omega-3 fatty acids increased adiponectin levels^16^. Dietary intake of magnesium has also been found to be associated with increased adiponectin levels in the general population^17,18^. In addition to omega-3 fatty acids and magnesium, cereal fibre contained in vegetables is also associated with adiponectin levels. Qi *et al*. found that a high intake of cereal fibre was associated with increased plasma adiponectin levels after adjusting for lifestyle factors, and that adiponectin levels were 19% higher in the highest quintile of cereal fibre intake than in the lowest quintile^18^. Furthermore, a high-fat diet has been found to decrease adiponectin and AdipoR2 mRNA expression^19^. Precarious workers tend to have a lack of routine and unhealthy dietary habits^2^. Unhealthy dietary habits are associated with low levels of omega-3 fatty acids, magnesium, and cereal fibre intake, and a high-fat diet, all of which lead to lower adiponectin levels.

A second possible reason for the effect of employment status on adiponectin levels, is that stress might be linked to adiponectin levels^19,20^. According to de Oliveira *et al*.^19^, an excess of glucocorticoids decreases adiponectin levels^19^. In one study, precarious work has been associated with high stress levels^4^. Thus, precarious workers experience high levels of stress, which can lower adiponectin levels. For these reasons, precarious workers may have lower adiponectin levels, compared with permanent workers. However, in our study, there was an association between precarious work and lower adiponectin levels, even after adjusting for job stress and psychological stress. In addition to job stress and psychological stress, other unmeasured stress may be related to lower adiponectin levels. Future studies should examine the relationship between adiponectin levels and types of stress that were not measured in this study.

A possible reason for our finding that the adiponectin levels in male precarious workers and in female precarious workers with low annual household incomes were significantly lower than those in permanent workers might be related to gender-related differences in social roles. Employment status has different effects on health of men and women^21^. In Japan, the concept of precarious work may differ by sex because of gender-related differences in the effect of job insecurity on psychological stress^22^. A previous study found that men with insecure jobs may be more stressed than those with secure jobs^22^. Considering the role of man as a breadwinner, there may be an association between precarious work and the level of stress. The primary role of many middle-aged women is considered to be a caregiver mother. Women in low-income households may need to take on precarious work in order to maintain an adequate household income. This may lead to a life-work imbalance and a higher level of stress.

Some other studies have had findings that contrast with our findings^15^. In a study of 4340 African-Americans, men of low socioeconomic status had significantly higher levels of adiponectin than men of high socioeconomic status. The difference in findings between this previous study and our study might be attributable to differences in the age and ethnicity of the study participants. Firstly, natriuretic peptide, which varies according to age, enhances adiponectin production in human adipocytes^23^, and is a modulator or confounder of adiponectin^13^. Natriuretic peptide levels increase with age^24^. The participants in our study had a mean age of 38 years, compared to a mean age of 55 years among the participants in the study of African-Americans, and so they might have had lower natriuretic peptide levels on average. Secondly, the baseline adiponectin level may vary by ethnicity, and may be higher in people of European origin than in African-Americans^25^. It is possible that there are also ethnicity-related differences in baseline adiponectin levels between African-Americans and the Japanese.

Because precarious workers, as a group, have a high risk of cardiovascular disease as well as a tendency towards lower adiponectin levels, various intervention programmes need to be implemented in this population. Precarious workers should be screened for modifiable risk factors, including obesity, hypertension, diabetes, diet, and job stress. They should be supported in their efforts to increase their adiponectin levels by encouraging them to adopt healthy dietary habits such as the consumption of omega-3 fatty acids, in order to prevent cardiovascular disease. In our study, we found that precarious work was associated with lower levels of adiponectin, even after adjusting for household income and level of education. This suggests that, the cause of their low adiponectin levels may not have been a lack of knowledge or economic access, but rather their degree of freedom with respect to the use of time. In view of this, environmental interventions may be more effective than educational activities. It is desirable that to create workplace and local environments that facilitate changes among precarious workers towards adapting a healthier lifestyle.

There were several limitations to our study. Firstly, the study had a cross-sectional design, and so we are unable to make causal inferences. Prospective study designs should be considered in further research on relationships between employment status and adiponectin levels in order to provide more insight regarding the causal direction of the association. Secondly, the findings of this study cannot be generalised to other ethnic or age groups. Thirdly, although cumulative evidence has shown that lower adiponectin levels increase the risk of metabolic diseases and arteriosclerosis, recent studies have found that higher adiponectin levels are associated with higher mortality^13,14^. However, contrary to the findings of recent epidemiological studies, Mendelian randomization studies have not found evidence that adiponectin plays a role in the causation of cardiovascular disease^26–28^. In our study, we did not have information on measured variation in genes. This is an important area for examination in future work. Fourthly, there may have been misclassification with regard to employment status, because participations self-reported their employment status. According to the results of a recent national labour survey in Japan, about 75% of people (about 90% of men, and 45% of women) aged 35 to 44 years are permanent workers^29^. In our study, 70.0% of participants, 79.7% of the male participants, and 36.7% of the female participants, were permanently employed. The similarity between the national figures regarding employment status, and the figures in our study, suggests that the classification of employment status among participants in our study was valid. Lastly, further studies are needed to assess whether other factors, such as nutrients in the diet, and other forms of stress, may explain the association between employment status and adiponectin.

## Conclusion

The study found that, among Japanese workers, male precarious workers and their female counterparts with low annual household incomes had significantly lower levels of adiponectin, compared with permanent workers. These findings can help provide an understanding of one of the mechanisms underlying the relationship between employment status and the onset of cardiovascular disease. Further studies are needed to assess whether changes in employment status are associated with changes in adiponectin levels.

## Methods

### Data source

We conducted a secondary data analysis using data from the Japanese Stratification, Health, Income, and Neighborhood (J-SHINE) study, a population-based survey conducted in metropolitan cities in Japan. The details of this survey have been described previously^30^.

Briefly, in order to investigate associations between socioeconomic status and health conditions, a probability sample of community-dwelling adults of working age (25–50 years) was drawn from the 2010 residential registration records. Participants answered a self-administered questionnaire using a computer-assisted self-interview programme. Of those who were invited, 4357(31.3%) agreed to participate. These participants formed the baseline of the J-SHINE study. In 2012, 2961 of the participants (69.0%) were followed up and of them, 2468 agreed to having their blood pressure measured, and 1205 agreed to have their blood chemistry measured. This is described in detail below.

### Demographics and socioeconomic conditions

Age, sex, and education level were self-reported in the 2010 survey. Participants’ age and sex were confirmed by checking residential registration records. Education level was categorised into two groups: ≤12 years or >12 years of formal education. Annual household income was assessed at baseline and follow-up using a self-administered questionnaire. In this study, we used the data from the 2012 follow-up survey. Each participant was asked to report their household income by selecting from a list 17 income categories. For this analysis, these levels were collapsed into three categories of annual household income: <2,999,000, 3,000,000–7,499,000, and ≥7,500,000 JPY. Marital status at the time of the survey was categorised as married or unmarried. The unmarried category included those who had never married, were divorced, and those who were widowed.

### Employment status

Employment status was assessed in each wave based on a self-administered questionnaire. In this study, we limited the sample to those with any paid employment. Each participant was asked to report their employment status by selecting from a list of nine employment status categories. For the analysis, employment status was classified as ‘permanent’, ‘precarious’, or ‘other’. Here, ‘permanent’ included managers, directors and full-time employees; ‘precarious’ included temporary employees, contract workers, and those with part-time jobs; and those who were self-employed, worked in a family business, had a private source of income, or did not provide an employment status, were classified as having an ‘other’ employment status. Of the participants, 70.0%, including 79.7% of the male participants and 36.7% of the female participants, were classified as permanent employees.

### Job stress

We used effort-reward imbalance (ERI) as an indicator of job stress^31^. ERI was assessed using a self-administered Japanese short version of the Effort-Reward Imbalance Questionnaire. The short version of the ERI Questionnaire consists of two scales, assessing ‘effort’ and ‘reward’. Effort was assessed using three items, and reward was assessed using seven items. Higher ratios of effort-to-reward correspond to higher levels of job stress. ERI was used as a continuous variable for the regression analyses.

### Psychological stress

We also used the Kessler Psychological Distress Scale (K6) as an indicator of psychological stress^32^. K6 is a six-item self-reported questionnaire designed to screen for mood and anxiety disorders. A cut-off value of five, which is what we used in this study, is used to screen for disease. The K6 score was divided into two categories for the regression analyses.

### Lifestyle

In the analysis, we included self-reported smoking status and physical exercise as lifestyle-related behaviours which may be related to obesity and visceral fat composition. Smoking status was classified as ‘never smoked’, ‘former smoker’, or ‘current smoker’ Physical exercise was classified as ‘ no exercise’, ‘ light exercise more than once a week’, ‘heavy exercise once or twice a week’, or ‘heavy exercise more than three times a week’.

### Biomarker measurements

Participants measured their waist circumference with paper tape, in front of investigator, who checked that the participants measured their waist circumference at navel height. Waist circumference was used as a continuous variable for the regression analyses. Systolic blood pressure (SBP) and diastolic blood pressure (DBP) were measured using a wrist blood pressure monitor with an advanced positioning sensor (Omron HEM-6371T, Omron Healthcare, Kyoto, Japan). The mean of three consecutive measurements was used for the analysis. The participants were invited to have their blood-chemistry measured at a convenient time. These measurements included serum lipid levels (low-density lipoprotein cholesterol [LDL-C] and high-density lipoprotein cholesterol [HDL-C]), haemoglobin A1c (HbA1c), high-sensitivity C-reactive protein, and adiponectin. Participants collected samples of their blood using a self-administered finger-prick blood sampling kit (Demecal Kit; Leisure, Inc., Tokyo, Japan). HbA1c values were standardised according to the National Glycohemoglobin Standardization Program. The plasma concentration of adiponectin was evaluated using a latex particle-enhanced turbidimetric immunoassay with an automated analyzer (Adiponectin Latex Kit, Otsuka Pharmaceutical Co., Ltd., Tokyo, Japan). The inter-assay coefficient of variation was less than 10.0%, and tests of stored specimens have found no biological degradation, indicating high validity of the measurements^33^.

### Ethical issues

Written informed consent was obtained from all participants. The study protocol and informed consent procedure were approved by the ethics committee of the Graduate School of Medicine of the University of Tokyo, and the study complied with the guidelines of the Declaration of Helsinki.

### Statistical analysis

**Figure 2:**
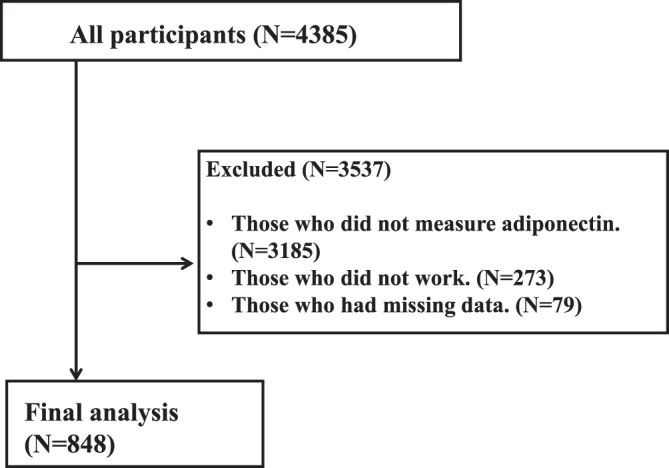
Flowchart showing the inclusion and exclusion criteria for this study.

Among those who agreed to have their blood chemistry measured, we excluded those who did not work (N = 273) and those with missing data (N = 79) from the analysis. The data from the remaining 848 individuals were used in this analysis (Fig. 2). We used log-transformed values of adiponectin, given its skewed distribution. As adiponectin values vary by sex, we stratified the analyses by sex. Differences in the independent variables were expressed as means with standard deviations, or as percentages. A one-way analysis of variance (ANOVA) was used to test for differences in the log-transformed adiponectin values by employment status. The Bonferroni correction was used to investigate the differences in adiponectin levels for each employment status. Multiple regression analysis was used to calculate the standardised coefficients (β) by the log-transformed adiponectin differences after adjusting for age, waist circumference, systolic blood pressure, marital status, smoking status, physical exercise, years of education, annual household income, job stress, psychological stress, and blood chemistry (LDL-C, HDL-C, and HbA1c). We conducted additional ad hoc stratified analyses by annual income level. A P-value < 0.05 was considered as statistically significant in the regression analyses, and a P-value < 0.1 was considered as statistically significant in the interaction. All analyses were performed using SPSS 21.0 J computer software for Windows (IBM SPSS Japan Inc., Tokyo, Japan).

## Data Availability

We were given permission to use the data from the J-SHINE study by the J-SHINE committee. The datasets used for this analysis are not publicly available, as the use of data from the J-SHINE study requires the permission of the J-SHINE committee.
